# Idiopathic Midaortic Syndrome: An Unusual Case of Acute Kidney Injury (AKI) in Secondary Hypertension With Renal Collaterals in an Adult

**DOI:** 10.7759/cureus.115530

**Published:** 2026-08-31

**Authors:** Rishikesh Kamble, Rajesh Jhorawat, Manish Chaturvedy, Vikrant Verma, Taruna Yadav

**Affiliations:** 1 Nephrology, All India Institute of Medical Sciences Jodhpur, Jodhpur, IND; 2 Pathology, All India Institute of Medical Sciences Jodhpur, Jodhpur, IND; 3 Radiology, All India Institute of Medical Sciences Jodhpur, Jodhpur, IND

**Keywords:** abdominal aortic occlusion, acute kidney injury, hemodialysis, mid-aortic syndrome, renal collateral circulation, secondary hypertension

## Abstract

Midaortic syndrome (MAS) is a rare vascular disorder characterized by segmental narrowing of the distal thoracic or abdominal aorta, accounting for only 0.5-2% of aortic coarctations. Adult-onset idiopathic MAS is particularly uncommon and typically presents with refractory secondary hypertension, whereas dialysis-dependent acute kidney injury (AKI) is an exceptionally rare manifestation. A 44-year-old man with a history of smoking and alcohol use presented with oliguria, vomiting, left flank pain, severe hypertension (210/110 mmHg), and advanced renal failure (serum creatinine 8.0 mg/dL). Laboratory investigations revealed mild proteinuria, microscopic hematuria, and thrombocytopenia, while autoimmune, infectious, and thrombotic evaluations were unremarkable. Renal biopsy demonstrated moderate acute tubular injury with minimal chronic changes. Despite initiation of hemodialysis and intensive antihypertensive therapy, the patient remained dialysis dependent. Careful re-examination identified diminished lower-limb pulses and a significant upper- to lower-limb blood pressure gradient. CT aortography demonstrated complete occlusion of the abdominal aorta distal to the superior mesenteric artery with extensive bilateral renal collateral circulation, consistent with idiopathic MAS. PET imaging excluded active large-vessel vasculitis, and the absence of significant atherosclerotic disease supported an idiopathic etiology. A repeat renal biopsy again showed acute tubular injury without significant fibrosis or hypertensive vascular changes. Given the preserved collateral renal perfusion, dialysis frequency was gradually reduced and ultimately discontinued. Over the subsequent two weeks, urine output increased to 1.5 L/day, accompanied by progressive recovery of renal function and resolution of volume overload. This case highlights that idiopathic MAS should be considered in adults presenting with refractory hypertension and unexplained dialysis-dependent AKI, particularly when pulse deficits or inter-limb blood pressure discrepancies are present. Extensive collateral renal circulation may preserve renal viability despite complete aortic occlusion. Recognition of this unique hemodynamic physiology may prevent unnecessary prolonged dialysis, as excessive ultrafiltration may compromise collateral-dependent renal perfusion and delay renal recovery.

## Introduction

Midaortic syndrome (MAS) is a rare vascular disorder characterized by narrowing of the distal thoracic and/or abdominal aorta, accounting for approximately 0.5-2% of aortic coarctations, and is idiopathic in nearly two-thirds of cases [1]. In children, MAS is commonly associated with genetic syndromes, including Alagille syndrome, neurofibromatosis type 1, and Williams syndrome, whereas in young adults, inflammatory and non-inflammatory arteriopathies such as Takayasu arteritis and fibromuscular dysplasia predominate [2,3]. In adults, Takayasu arteritis and atherosclerosis are among the leading acquired causes, with hypertension being the most frequent clinical manifestation [4]. The hemodynamic consequences of MAS depend on the severity and anatomical level of aortic narrowing. Proximal to the stenotic segment, increased vascular resistance and compensatory elevation of arterial pressure can result in severe hypertension, whereas distal to the obstruction, reduced aortic blood flow may lead to systemic and renal hypoperfusion. The renal arteries typically arise from the abdominal aorta around the level of the upper lumbar vertebrae; therefore, their relationship to the stenotic segment is critical in determining renal blood flow. When the renal arteries originate distal to or are involved by the stenosis, reduced renal perfusion may activate the renin-angiotensin-aldosterone system, further aggravating hypertension and potentially contributing to acute kidney injury (AKI). Collateral vessels, including mesenteric, lumbar, and other systemic arterial pathways, may partially compensate for reduced distal aortic flow and preserve renal and visceral perfusion. However, renal function may become vulnerable when collateral perfusion is inadequate or compromised, particularly during episodes of reduced effective circulating volume or excessive fluid removal. We report a rare case of adult-onset idiopathic MAS presenting as dialysis-dependent AKI, emphasizing the role of collateral circulation in preserving renal viability and the potential pitfalls of aggressive fluid removal.

## Case presentation

Demographics and initial presentation

A 44-year-old man, a chronic smoker and alcohol user, presented with left flank pain, vomiting, and oliguria for seven days. Newly diagnosed hypertension with no previous history of antihypertensive medications. There was no known family history of hypertension, renal disease, or cardiovascular disease. He denied hematuria, edema, seizures, headache, or visual disturbances. On examination, his blood pressure was 210/110 mmHg, with severe hypertensive retinopathy on fundoscopy. Importantly, there was a significant upper- to lower-limb blood pressure (BP) gradient of right upper limb BP 210/100, left upper limb BP 200/110 mmHg, right lower limb BP 120/90, and left lower limb BP 120/86 mmHg with diminished peripheral pulses in the lower limbs. These findings raised the possibility of an underlying renovascular or large-vessel disorder. Ultrasonography showed bilaterally normal-sized kidneys with preserved corticomedullary differentiation (Figure 1A, 1B).

**Figure 1:**
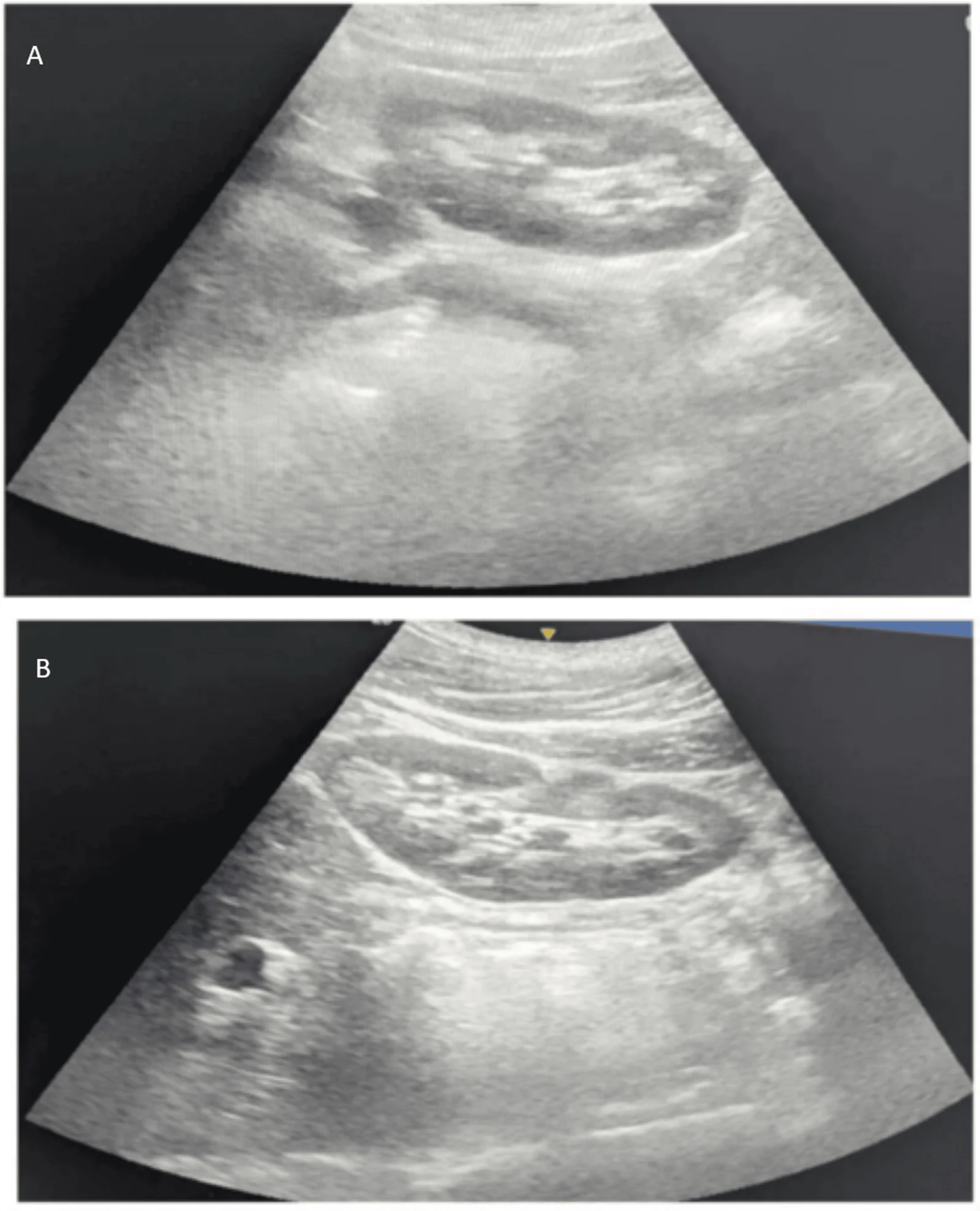
Ultrasound Imaging of Kidneys (A) Right kidney with preserved corticomedullary differentiation (B) Left kidney with preserved corticomedullary differentiation

Initial investigations

Laboratory investigations revealed advanced renal failure, with a serum creatinine of 8.0 mg/dL, along with thrombocytopenia. Urinalysis showed 3+ proteinuria, 10-12 RBCs/high power field (hpf), and 40-45 pus cells/hpf. However, 24-hour urinary protein excretion was only 287 mg/day, and urine culture was sterile. An extensive evaluation for autoimmune, infectious, and thrombotic causes was unrevealing. Antinuclear antibody (ANA), antineutrophil cytoplasmic antibody (ANCA), anti-glomerular basement membrane (anti-GBM) antibodies, complement levels, HIV, hepatitis B surface antigen (HBsAg), and hepatitis C virus (HCV) were negative. Peripheral blood smear showed no schistocytes. ECG demonstrated left ventricular hypertrophy. The relevant laboratory findings are summarized in Table 1.

**Table 1:** Baseline Investigation ANA: antinuclear antibody, ANCA: antineutrophil cytoplasmic antibody, anti-GBM: anti-glomerular basement membrane, HBsAg: hepatitis B surface antigen, HCV: hepatitis C virus, HPF: high-power field, GFR: glomerular filtration rate, CKD-EPI: Chronic Kidney Disease Epidemiology Collaboration, ESR: erythrocyte sedimentation rate, CRP: C-reactive protein

Investigation	Result	Reference Range
Hemoglobin	11 g/dL	13.0-17.0 g/dL
Total leukocyte count	14×10⁹/L	4.0-11.0 ×10⁹/L
Platelet count	Thrombocytopenia (80×10⁹/L)	150-400 ×10⁹/L
Peripheral blood smear	No schistocytes	Absent
Blood urea nitrogen (BUN)	202 mg/dL	7-20 mg/dL
Serum creatinine	8.0 mg/dL	0.7-1.3 mg/dL
Estimated GFR (CKD-EPI)	8 mL/min/1.73 m²	>90
Sodium	128 mmol/L	135-145 mmol/L
Potassium	4.8 mmol/L	3.5-5.0 mmol/L
Chloride	100 mmol/L	98-107 mmol/L
Bicarbonate	14 mmol/L	22-28 mmol/L
Calcium	9.1 mg/dL	8.5-10.5 mg/dL
Phosphate	4.9 mg/dL	2.5-4.5 mg/dL
Urine Protein	3+	Negative
Urine Red blood cells	10-12/HPF	0-2/HPF
Urine White blood cells (Pus cells)	40-45/HPF	0-5/HPF
24-hour urinary protein	287 mg/day	<150 mg/day
Urine culture	Sterile (No growth)	Sterile
ANA	Negative	Negative
ANCA	Negative	Negative
Anti-GBM antibody	Negative	Negative
Complement C3	98 mg/dL	90-100 mg/dL
Complement C4	30 mg/dL	10-40 mg/dL
HIV antibody	Negative	Negative
HBsAg	Negative	Negative
Anti-HCV antibody	Negative	Negative
ESR	56 mm/h	<20 mm/h
CRP	136 mg/L	<5 mg/L

Initial management

Hemodialysis was initiated for severe renal failure (Kidney Disease: Improving Global Outcomes (KDIGO) Stage 3 AKI) and oliguria, while intravenous nitroglycerin was administered for blood pressure control. A renal biopsy was performed to evaluate the cause of the AKI (KDIGO stage 3) as there was no improvement in renal function despite adequte blood pressure control. Histopathological examination demonstrated moderate acute tubular injury with minimal interstitial fibrosis and tubular atrophy (5-10%) (Figure 2A, 2B), without significant chronic glomerular or vascular changes.

**Figure 2:**
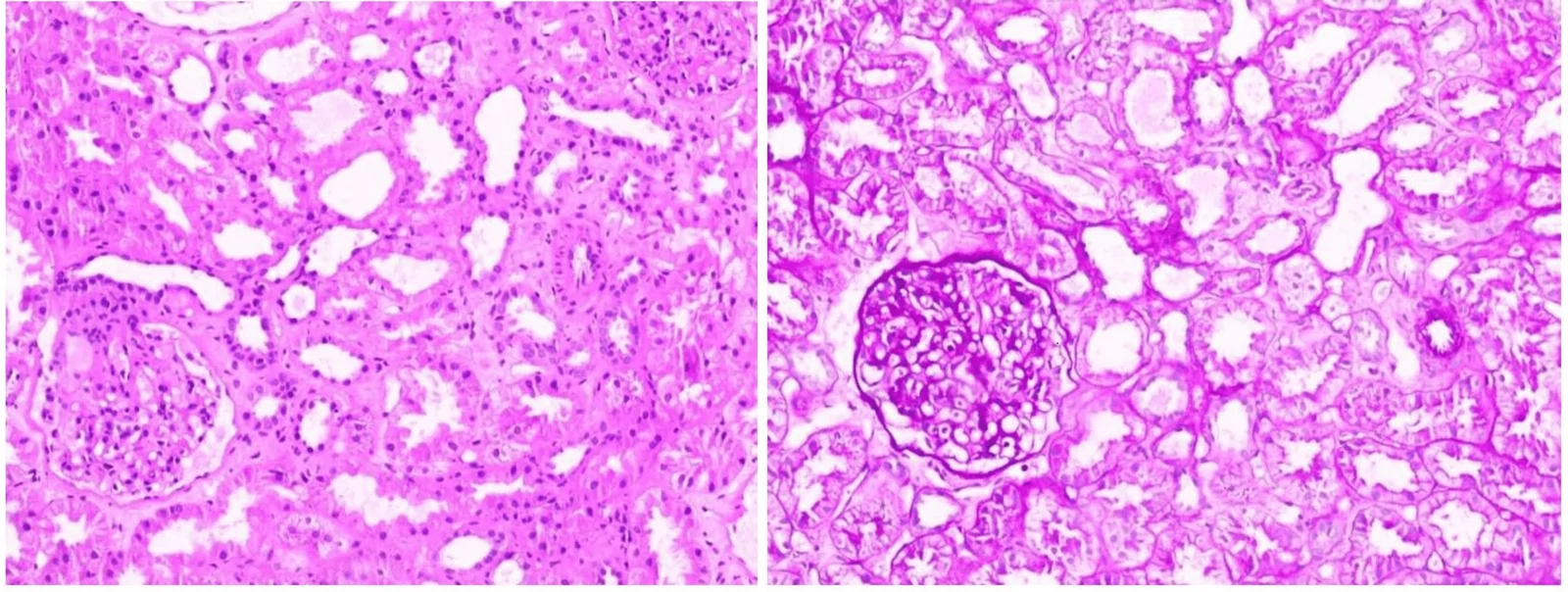
Histopathology (A) First kidney biopsy (KBx): Images showing acute tubular injury in the form of luminal dilatation and simplification of the lining epithelium. Glomeruli are largely unremarkable. (B) Second KBx showing acute tubular injury in the form of luminal dilatation and simplification of the lining epithelium. Glomeruli are largely unremarkable. No increase in interstitial fibrosis and tubular atrophy in second biopsy (A- H&E, x 100; B- Periodic acid-Schiff stain, x100).

Diagnostic re-evaluation

Despite four weeks of hemodialysis and intensive antihypertensive therapy, the patient remained dialysis-dependent, with persistent hypertension and no significant recovery of renal function. In view of the severe hypertension, lower-limb pulse deficits, and marked upper- to lower-limb blood pressure gradient, the possibility of a large-vessel vascular disorder was reconsidered. CT aortography demonstrated complete occlusion of the abdominal aorta distal to the superior mesenteric artery, with extensive bilateral renal collateral circulation (Figure 3A-3C), consistent with MAS. PET imaging showed no evidence of active large-vessel vasculitis. In the absence of significant atherosclerotic disease and other identifiable secondary causes, an idiopathic etiology was considered.

**Figure 3:**
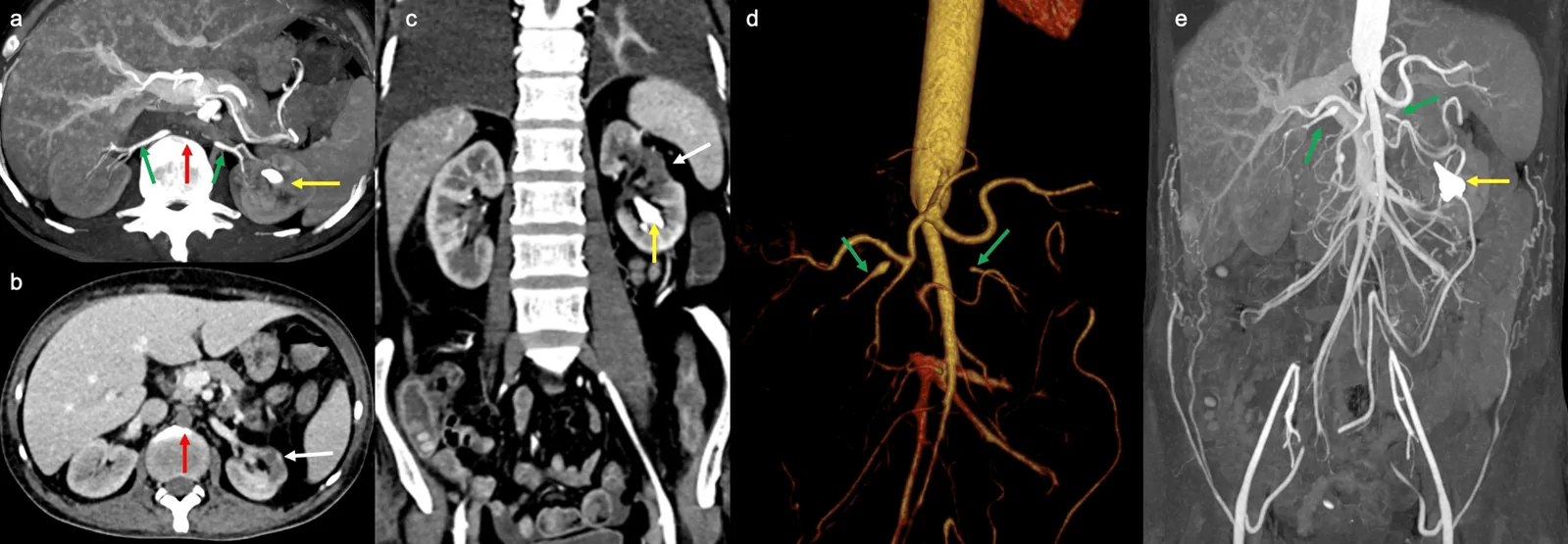
Radiological Imaging Dual-phase CT angiography images. (A) Axial maximum intensity projection (MIP) images of the arterial phase and (B) Venous phase showing aortic thrombosis (red arrows) below the origin of the superior mesenteric artery with contiguous thrombotic occlusion of bilateral renal arteries at the ostium and proximal parts. Bilateral main renal arteries are reformed by collaterals (green arrows in A, D, E). (C) Coronal reformat depicting a left upper-pole cortical infarct and scarring (white arrows in B, C) along with a large calculus in the left lower calyceal (yellow arrows in A, C, E). Bilateral kidneys show relatively preserved enhancement. (D) 3D volume rendered, (E) coronal MIP images showing complete aorto-iliac thrombosis with reformation of bilateral iliac arteries via collaterals.

Intervention and treatment modification

Given the extensive collateral circulation supplying the kidneys and the absence of significant irreversible renal injury on biopsy, renal perfusion was considered potentially adequate for recovery. Dialysis frequency was therefore progressively reduced, with careful avoidance of excessive ultrafiltration and maintenance of adequate intravascular volume. Antihypertensive therapy was continued with close monitoring of blood pressure and volume status. A repeat renal biopsy demonstrated persistent acute tubular injury without significant fibrosis or hypertensive vascular changes, further supporting the potential reversibility of the renal injury.

Outcome

Over the subsequent two weeks, Dialysis frequency was therefore reduced and eventually stopped. Over the next two weeks, urine output improved to 1.5 L/day with a reduction in weight and edema, indicating recovery of renal function (Figure 4A, 4B). The clinical course suggested recovery from potentially reversible ischemic acute tubular injury supported by extensive collateral renal perfusion despite complete abdominal aortic occlusion.

**Figure 4:**
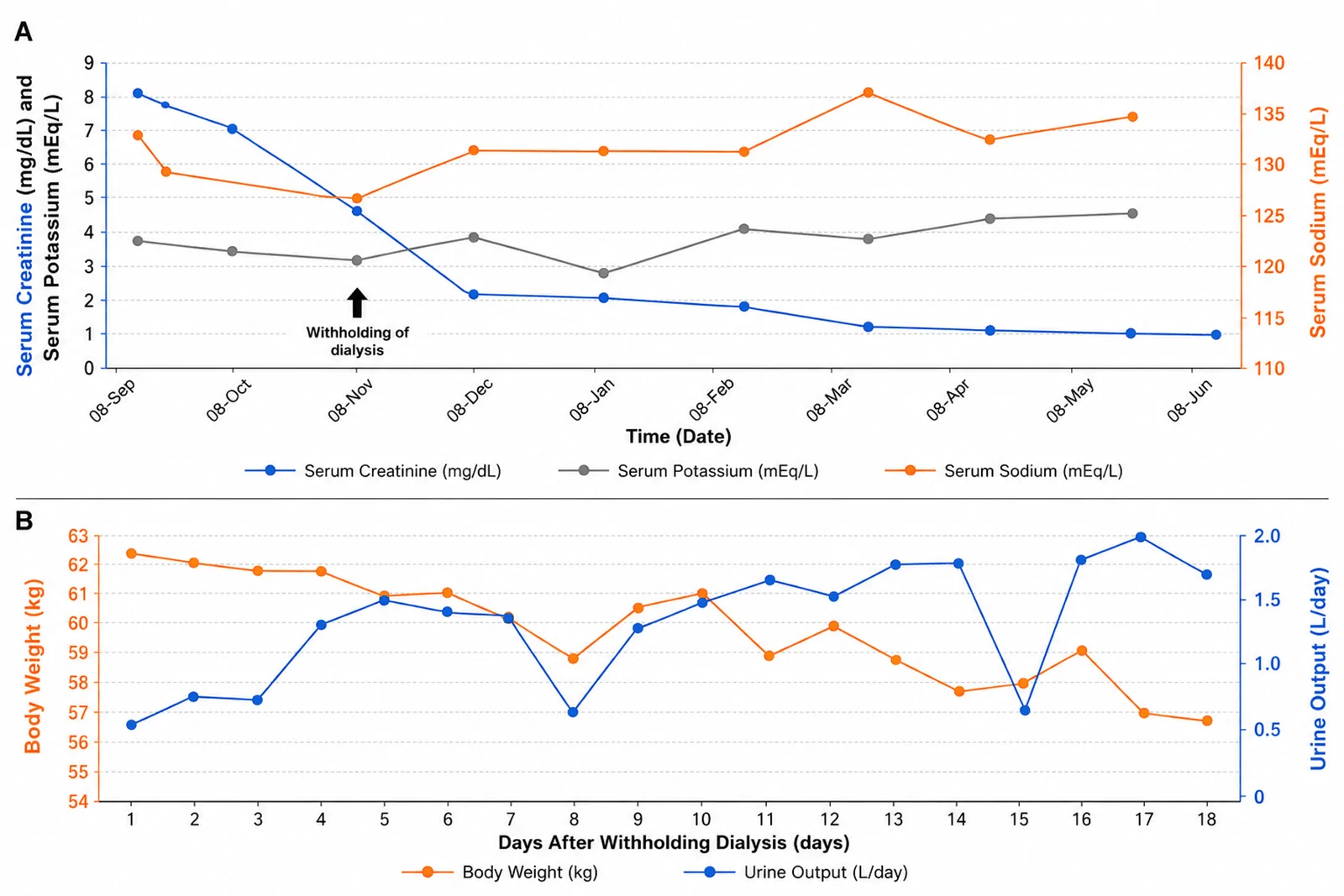
Graphical representation of improvement of kidney function and urine output (A) Graph showing the change in serum creatinine, potassium, and sodium before and after withholding of dialysis. The black arrow indicates the time to withhold dialysis. The blue line indicates the trend of serum creatinine (mg/dl), the orange line indicates the trend of serum sodium (mEq/L), and the grey line indicates the trend of serum potassium (mEq/L). Both serum creatinine and potassium are represented on the left-sided Y-axis (0-9). Serum sodium is represented on the right-sided Y-axis (110-140). The X-axis represents days common to all three parameters. (B) Graph showing change in urine output (UO in liters) and body weight (Bwt in kgs) after withholding dialysis. The left-sided Y-axis represents body weight (from 54 to 63 kg), and the right-sided Y-axis represents urine output (UO) in liters. The blue line represents UO, and the orange line represents body weight. The X-axis represents days after withholding of dialysis.

## Discussion

Diagnosis of idiopathic MAS

Our patient, a 44-year-old chronic smoker and alcohol consumer, had no constitutional symptoms and sparing of the subclavian arteries. A normal PET scan excluded large-vessel vasculitis, making Takayasu arteritis unlikely. Although atherosclerotic renovascular disease is common in smokers, typical atheromatous plaques and vascular calcification were absent (Figure 3A-3E). Idiopathic MAS, the most frequently reported form, was therefore considered the most likely diagnosis. Renal artery involvement in MAS commonly causes hypertension and predisposes to AKI. In this case, left-sided nephrolithiasis with vomiting and hypertension likely precipitated prerenal AKI, progressing to intrarenal injury requiring dialysis. A focal left cortical infarct likely explained the albuminuria and hematuria, while renal perfusion was preserved by extensive bilateral collateral circulation (Figure 3C).

Renal collateral and its origin

Renal collateralization develops through angiogenesis or arteriogenesis. Angiogenesis involves sprouting of new capillaries from existing microvasculature and is markedly stimulated in ischemic kidneys by oxidative stress and inflammation. Key mediators such as VEGF, HIF-1α, and angiopoietins promote increased capillary density. However, these vessels are typically small, fragile, and limited in conductance capacity. Although they enhance local microcirculatory perfusion, they are generally insufficient to restore normal renal blood flow in the setting of major arterial obstruction, making this mechanism less likely in our case [5,6]. Arteriogenesis, in contrast, refers to the remodeling and enlargement of preexisting arteriolar connections into functional collateral arteries. This process is primarily driven by increased shear stress and inflammatory cytokines, including MCP-1 and TNF-α [7-9]. Progressive luminal expansion allows substantial augmentation of blood flow, ultimately forming a mature, hemodynamically efficient collateral network that best explains our findings.

Impact of ultrafiltration on collateral renal perfusion

Collateral vessels may protect the kidney from overt ischemia but cannot fully restore normal renal physiology. In our patient, renal size was preserved, yet persistent severe hypertension, oliguria, dyspnea, and progressive weight gain required continuation of hemodialysis with ultrafiltration (2-2.5 L per session). Despite regular fluid removal, weight gain continued, likely reflecting ongoing renin-aldosterone-mediated sodium and water retention. A repeat renal biopsy again demonstrated acute tubular injury without hypertensive vascular or glomerular changes, suggesting that repeated ultrafiltration may have contributed to delayed renal recovery. Previous studies have also shown that ultrafiltration can adversely affect urine output and clinical outcomes in dialysis-dependent patients [10]. Thus, a vicious cycle of oliguria, refractory hypertension, and aggressive ultrafiltration may have perpetuated his clinical instability. Although discontinuing dialysis after more than four weeks of AKI was challenging, the absence of significant fibrosis on repeat biopsy supported a trial of withdrawal. Following cessation of dialysis, urine output improved, body weight declined, and both clinical and biochemical parameters showed recovery.

## Conclusions

This case highlights MAS as an important and potentially reversible cause of secondary hypertension, which can present as rapidly progressive renal failure in adults. While well-developed renal collaterals may preserve cortical perfusion, excessive ultrafiltration can aggravate renin-aldosterone activation, worsen hypertension, and delay renal recovery in AKI.
